# The effectiveness of anti-interleukin-1 therapy on subclinical inflammation parameters during the attack-free period in familial Mediterranean fever patients: A case-control study

**DOI:** 10.3906/sag-2106-324

**Published:** 2021-12-25

**Authors:** Ebru ATALAR, İsmail DOĞAN, Kevser GÖK, Serdar Can GÜVEN, Yüksel MARAŞ, Gülay Güleç CEYLAN, Sükran ERTEN

**Affiliations:** 1 Division of Rheumatology, Department of Internal Medicine, Ankara City Hospital, Ankara, Turkey; 2 Department of Medical Genetics, Ankara City Hospital, Ankara, Turkey

**Keywords:** Amyloidosis, antiinflammatory agents, anti-IL-1 therapy, biological therapy, familial Mediterranean fever, inflammation

## Abstract

**Background/aim:**

Anti IL-1 therapy is useful in suppressing attacks in FMF patients with colchicine resistance, however, it is not certain whether subclinical inflammation can sufficiently be inhibited with anti-IL-1 therapy in FMF patients with amyloidosis.

**Materials and methods:**

Forty-six FMF patients receiving anti-interleukin-1 therapy and 36 healthy control patients were compared in terms of laboratory parameters. Also, FMF patients were further divided into two groups; those with amyloidosis and those without it, and these subgroups were compared to each other in terms of clinical and laboratory findings.

**Results:**

In comparison between the FMF and healthy control groups, erythrocyte sedimentation rate (ESR), C-reactive protein (CRP) level, and red cell distribution width (RDW) level were detected to be higher and hemoglobin level lower in the patient group. Within the FMF patient group, the ESR, CRP, fibrinogen, RDW, and NLR values were significantly higher in the subgroup with amyloidosis in comparison to the subgroup without amyloidosis.

**Conclusion:**

Anti-interleukin-1 therapy could not fully suppress the subclinical inflammatory parameters when compared to healthy individuals.

## 1. Introduction

Familial Mediterranean fever (FMF) is an autosomal recessive disorder characterized by recurrent febrile episodes and serosal inflammation lasting for 2 to 3 days and resolving spontaneously [1]. It is a common disease in the Mediterranean region and most prevalent among people of Jewish, Turkish, Armenian, and Arabic ancestry [2]. Painful symptoms of serositis accompanying fever are typical of FMF. Ninety-five percent of the patients have abdominal attacks, while approximately 50% to 75% have attacks of arthritis that mainly affect the large joints of the lower limb [1]. Pleural inflammation, which occurs in the form of unilateral chest pain, is experienced in some patients. Pericarditis is rare and occurs in less than 1% of the patients with FMF [3].

To date, many inflammatory markers have been studied in FMF. The fibrinogen, erythrocyte sedimentation rate (ESR), C-reactive protein (CRP), serum amyloid A (SAA) protein, and white blood cell (WBC) levels are all used as markers of acute phase response (APR) in FMF [4]. These markers increase during the attacks and then usually return to normal in the attack-free period [5].

It is now known that subclinical inflammation may continue in attack-free periods of FMF patients [5–7]. This type of inflammation may lead to the development of amyloidosis, which is the most devastating complication of FMF. Amyloidosis is very frequent despite colchicine treatment and is the most important finding that determines the prognosis of FMF disease [8].

The neutrophil-to-lymphocyte ratio (NLR) is an index obtained from the proportion of neutrophils to lymphocytes. In recent studies, NLR has been a useful indicator of inflammation [5, 9].

Red cell distribution width (RDW) is included in a complete blood cell count panel and associated with the heterogeneity of red blood cells. Lippi et al. showed a strong association of RDW with CRP and ESR in their study [10]. Amyloidosis, an inflammatory disease, can cause RDW levels to increase.

Mean platelet volume (MPV) may also be an indicator of subclinical inflammation. However, contradictory results between MPV and subclinical inflammation have been reported [11–14].

The MEFV gene encodes the protein pyrin, essential in the innate immune system and inflammasome. Mutations in the MEFV (MEditerranean FeVer) gene are useful for the diagnosis of FMF [15]. It has been demonstrated that patients with FMF may present with subclinical inflammation even during the attack-free periods [15].

A macromolecular complex, called inflammasome, plays a major role in the activation of interleukin-1 **(**IL-1) and thus in the induction of inflammation, and when inflammasome activity is abnormally stimulated through a mutation, IL-1 may be involved in the pathogenesis of FMF [7,8]. Anti-interleukin-1 (anti-IL-1) therapy is used due to its efficacy in suppressing inflammation in FMF patients. Although the long-term efficacy and safety of anti-IL-1 therapy are not well known, it is used in the cases resistant to colchicine treatment due to its effectiveness in FMF complications [16]. Although the efficiency of these drugs is significant in the clinical picture, their effect in preventing subclinical inflammation has not been fully revealed yet.

In our study, we aimed to evaluate the clinical, biochemical, hematological, and genetic parameters that may be associated with subclinical inflammation in FMF patients (with amyloidosis and without amyloidosis) receiving IL-1 antagonist therapy.

## 2. Materials and methods

This study was planned as a case-control study. The study group consisted of patients diagnosed with FMF according to the Tel-Hashomer criteria [17] who were on follow-up at our clinic and were in the attack-free period. All patients had classical (Type 1) FMF, characterized by recurrent typical febrile attacks. IL-1 antagonist therapy was added to the colchicine treatment for patients who failed to give a sufficient response to colchicine treatment or who could not take efficient colchicine doses due to drug intolerance.

All patients in the study group were under either anti-IL-1 therapy or anti-IL-1 therapy + colchicine treatment and were in an attack-free period. An attack-free period was described as at least two weeks from the end of an FMF attack, based on the symptoms. Patients with one of the following conditions were excluded: 1) being in the active phase of the disease, 2) concomitant infections and/or inflammatory diseases such as ankylosing spondylitis, 3) chronic diseases such as diabetes mellitus, thyroid dysfunction, liver disease, atherosclerotic disease, chronic obstructive pulmonary disease (COPD), cerebrovascular diseases, 4) malignancy, 5) age under 20 years or above 75 years, 6) medications that could influence platelets and/or the coagulation system, such as aspirin and oral anticoagulants, 7) pregnancy, and 8) primary amyloidosis.

The median colchicine dose was 1 mg/day (range: 0.5 to 2 mg/day). Eleven patients subcutaneously received canakinumab (150 mg/month) while 35 patients took anakinra (100 mg/day). In three of the five patients who underwent hemodialysis, anakinra was administered three days a week. No dosage change was required for the two patients who took canakinumab.

For all patients, amyloidosis diagnosis was given by the histopathological examination of samples taken from the kidney, duodenum, rectum, bone marrow, or salivary gland. Amyloidosis was detected histologically with Congo red and immunohistochemically by AA staining.

Age- and sex-matched healthy subjects served as the control group. The control group was selected from the patients who were diagnosed with mechanical musculoskeletal pain in an outpatient clinic. The exclusion criteria applied to the patient group were also applied to the control group.

In the patient and control groups, laboratory features such as ESR, CRP, hemoglobin (Hb), WBC count, neutrophil, lymphocyte, platelet, MPV, and RDW levels were retrospectively retrieved from the hospital records. The NLR was calculated as a simple ratio between the absolute neutrophil count and absolute lymphocyte count. The FMF patient group was divided into two subgroups; those who had amyloidosis and those who did not. Their clinical and laboratory findings in addition to MEFV gene mutations were also recorded and compared.

The study protocol was approved by the Local Research Ethics Committee (no. E1/1543/2021) and conducted in accordance with the ethical principles described by the Declaration of Helsinki.

### 2.1. Sample size calculations

Based on the case-control study of Ahsen et al. [9], where the authors aimed to investigate the relationship between FMF and inflammation using the NLR, the effect size was calculated as d = 0.71, alpha error as 0.05, and 1-beta error as 0.80 using the mean and standard deviation values of the NLR (2.21 ± 0.86 and 1.68 ± 0.59, respectively), and it was determined that a total of 50 people (at least 25 people per group) were sufficient to test the null hypothesis. The G*Power v.3.1.9.4 (Universität Düsseldorf, Düsseldorf, Germany) statistics software was used for the analyses [18].

The continuous variables were expressed as mean ± standard deviation while the categorical data were presented as numbers and percentages. In the analysis of continuous variables between the groups, normality analyses were done using the Kolmogorov-Smirnov goodness of fit test. The t-test was used for the comparisons between two groups when the data were normally distributed, whereas the Mann-Whitney U test was employed for the nonnormally distributed data. Pearson’s correlation test was used in the correlation analysis and the chi-square test (Fisher’s exact test when appropriate) in the comparison of categorical data. The risk factors and estimated relative risk (odds ratio) values to determine the risk of amyloidosis in FMF patients were evaluated with the multivariate binary logistic regression, which was created by selecting the clinically significant variables in univariate analyses. The Hosmer-Lemeshow test was used for model fit. The cut-off values for the parameters were determined using the screening tests (sensitivity, specificity, PPD, NPD) and by ROC curve analysis. The analyses were performed using the IBM SPSS v.24.0 software (IBM Corp., Armonk, NY, USA). The statistical significance level was considered as p < 0.05.

## 3. Results

While the ESR, CRP, and RDW values in the patient group (11 [3–106], 3.12 [0.5–57] and 15 [12–21.5], respectively) were found to be statistically significantly higher in comparison to the control group (8 [3–38], 1.28 [0.5–8.6], and 13.3 [12.4–24], respectively) (p < 0.05), the Hb values were significantly lower in the patient group when compared to the control group (13.1 [8–17.3] vs. 14.5 [9–17.3]; p = 0.003). No statistically significant difference was detected between the patient and control groups in terms of WBC, neutrophil, lymphocyte, NLR, platelet, and MPV levels (p > 0.05) (Table 1).

**Figure 1:**
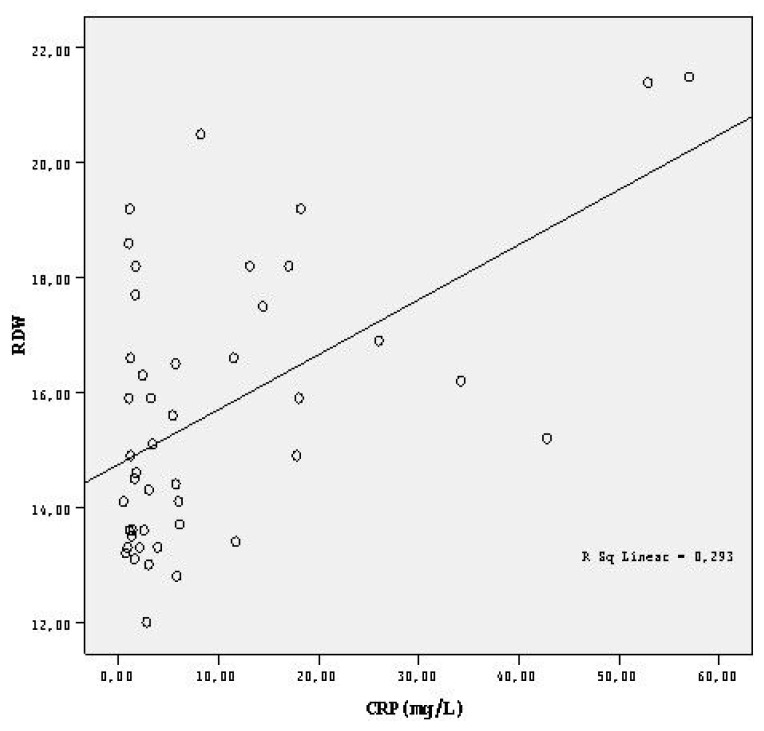
Graph showing the correlation between the CRP and RDW levels in FMF patients.

In FMF patients, a statistically significant, moderate/strong positive correlation was detected between the mean CRP and RDW values (p < 0.001, r = 0.541) (Table 2, Figure 1).

**Figure 2:**
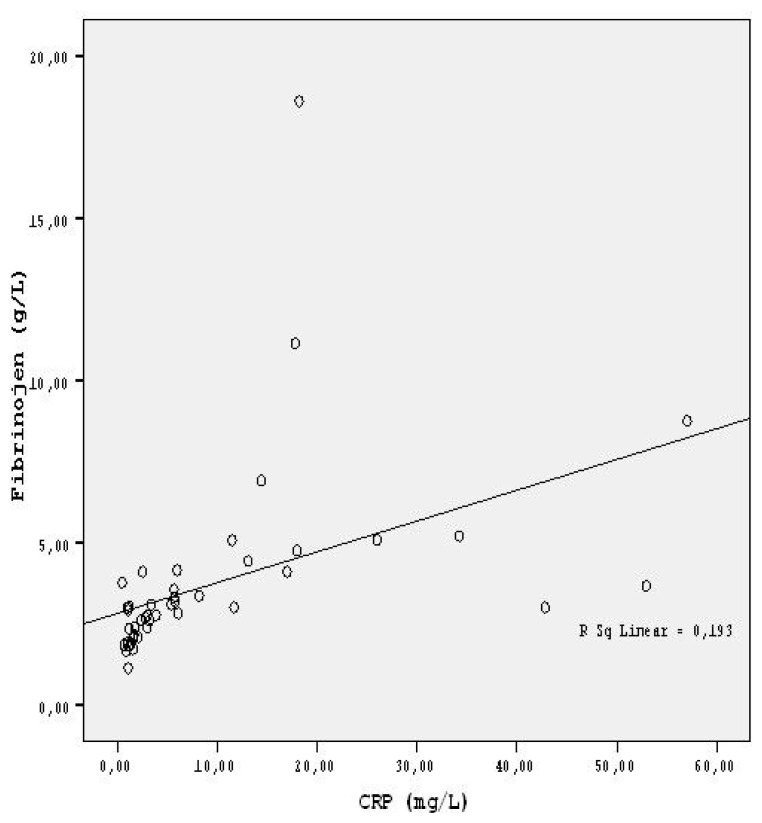
Graph showing the correlation between the CRP and fibrinogen levels in FMF patients.

A statistically significant, moderate positive correlation was detected between the mean CRP and fibrinogen means in FMF patients (p = 0.002, r = 0.439). (Table 2, Figure 2).

The duration of IL-1 antagonist therapy in FMF patients with amyloidosis was significantly higher than that in FMF patients without amyloidosis (69.5 months [range: 23 to 85 months] vs. 31 months [range: 5 to 84 months]; p = 0.005). While 80.0% (n = 16) of the FMF patients with amyloidosis were males, 80.8% (n = 21) of the patients without amyloidosis were females (p < 0.001). There was arthralgia/arthritis in 30.0% (n = 6) of the patients with amyloidosis, whilst these conditions were detected in 61.5% of the patients without amyloidosis (p = 0.042). No statistically significant difference was observed between the amyloidosis-positive and -negative patients in terms of age, BMI, age at the time of diagnosis, duration of the disease, family history, consanguinity history, genetic mutation (genetic test results of two patients could not be achieved), the frequency of fever, erysipelas, peritonitis, pleurisy, or myalgia, and the mean colchicine dose administered (p > 0.05) (Table 3).

In FMF patients with amyloidosis, the mean/median values of fibrinogen, ESR, CRP, WBC, neutrophil, NLR, and RDW were significantly higher than those without amyloidosis (p < 0.05). No statistically significant differences were observed between the amyloidosis-positive and -negative patients in terms of the mean/median values of lymphocyte count, platelet count, Hb, and MPV (p > 0.05) (Table 4).

According to the multivariate binary logistic regression analysis performed to determine the factors that increase the risk of amyloidosis in FMF patients, age, gender, fibrinogen, ESR, CRP, NLR, RDW, and the presence of arthralgia/arthritis variables, which were found to be clinically and/or statistically significant, were included in the model, while the ESR, male gender, and the presence of arthralgia/arthritis variables remained significant in the model (Table 5).

**Figure 3:**
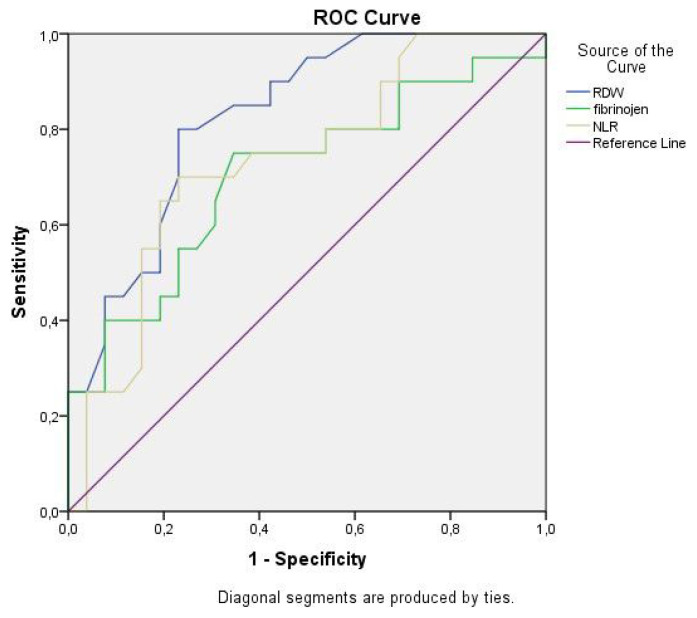
ROC curve analysis of RDW, fibrinogen, and NLR as a diagnostic marker for amyloidosis positivity in FMF patients.

The ROC curve analysis was done to see if fibrinogen, NLR, and RDW values could be used as a diagnostic marker for amyloidosis positivity in FMF patients. For fibrinogen, we found a cut-off value of ≥2.97, sensitivity of 75.00%, and specificity of 65.40%, while the positive predictive value was 52.20% and the negative predictive value was 47.80%. The area under the curve was calculated as 0.705, with a standard error of 0.080 (p = 0.018). For NLR, we found a cut-off value of ≥2.16, sensitivity of 70.00%, and specificity of 43.50%, while the positive predictive value was 77.00% and the negative predictive value was 56.50%. The area under the curve was calculated as 0.734, with a standard error of 0.075 (p = 0.007). For RDW, we found a cut-off value of ≥15.40, sensitivity of 75.00%, and specificity of 76.90%, while the positive predictive value was 45.70% and the negative predictive value was 54.30%. The area under the curve was calculated as 0.827, with a standard error of 0.060 (p < 0.001) (Table 6, Figure 3).

## 4. Discussion

Our results demonstrated, ESR, CRP and RDW levels were higher and hemoglobin levels were lower in FMF patients under anti IL 1 therapy when compared to healthy subjects. Within the FMF patient group, the ESR, CRP, fibrinogen, RDW, and NLR values were significantly higher in the subgroup with amyloidosis in comparison to the subgroup without amyloidosis.

Fibrinogen, ESR, CRP, SAA protein, and WBC values are used as laboratory parameters of APR in FMF. These laboratory parameters increase during the FMF attack and generally return to normal limits during the attack-free periods [4,5]. Some studies show that inflammation may continue during the attack-free periods in patients with FMF [5,19]. A permanent increase in acute-phase proteins during the attack-free periods is important because it reflects inflammation even in the absence of clinical findings. Persistent subclinical inflammation in FMF patients may increase the risk of amyloidosis, which can be fatal [5, 20]. Amyloidosis is the accumulation of proteins called amyloid protein in various tissues and organs in the body. There are different types of amyloidosis. Secondary (AA) amyloidosis is seen in FMF and it most frequently affects the kidney, heart, peripheral nerves, thyroid gland, and bone marrow. The most important predictors of survival in patients with FMF are heart and kidney involvement [20,21].

The role of the IL-1 cytokine is remarkable in autoimmune diseases such as FMF. The IL-1 cytokine refers to IL-1α and IL-1β cytokines [22]. This protein plays an important role in the production of acute-phase proteins and inflammation. The IL-1 receptor antagonist (IL-1Ra), naturally present in the body, controls the activity of IL-1 by competing with IL-1 to bind to the IL-1 receptor. Anakinra is a recombinant form of human IL-1 receptor antagonist that competitively inhibits the binding of IL-1α and IL-1β to the endogenous IL-1 receptor [23]. The other drug used in therapy, canakinumab, is a monoclonal antibody that is created against IL-1β and neutralizes its bioactivity [24,25]. These drugs, which are costly and require high technology, are used in the treatment of autoinflammatory diseases. If there is no response to treatment with other drugs in FMF patients, it is necessary to use these drugs [26].

In our study, the ESR, CRP, and RDW values in FMF patients were found to be significantly higher compared to the healthy population. We believe that these findings are related to inflammation [10]. On the other hand, Hb values were low. We can argue that anti-IL-1 therapy did not display sufficient impact on suppressing these parameters that indicated an inflammation during the attack-free periods. However, because our study is a case-control study and we have not followed up these parameters before and after IL-1 antagonist therapy, we did not have the opportunity to assess the efficacy of the drugs on this subject objectively.

Platelets are multifunctional blood cells and are considered to be effective in initiating inflammation and fibrosis, which are associated with a variety of prothrombotic and proinflammatory diseases [27]. Mean platelet volume is a marker of platelet function and activation [28]. A high level of MPV is considered a risk factor for myocardial infarction and ischemic cardiovascular disease [29,30]. The increased platelet count and function in inflammatory diseases can be explained by the stimulation of platelet release by proinflammatory cytokines, especially the IL-6. The course of the inflammatory response is also associated with the percentage of large platelets. The large platelets migrate to the inflamed area. Accordingly, MPV is expected to be low in ongoing inflammation [27]. Gasparyan et al. demonstrated that high-grade inflammatory diseases, such as attacks of FMF, present with low levels of MPV, which have increased due to the administration of antiinflammatory drugs [31,32]. On the contrary, according to Yazıcı et al., it has been asserted that high MPV values in rheumatoid arthritis patients are associated with disease activity [33]. In our study group, no difference could be found between the patients with FMF and healthy individuals in terms of platelet and MPV levels. Similarly, these two laboratory parameters were not different in FMF patients with amyloidosis and without amyloidosis.

Red cell distribution width is part of the hemogram and is accepted as the variability in the size of circulating erythrocytes [34]. Higher RDW values are accepted as a strong predictor of mortality in patients with heart failure and in adults 45 years or older [35,36]. Inflammation is considered to play a role in the etiopathogenesis of cardiovascular diseases [37,38]. According to the hypothesis of Tonelli et al., the relationship between high RDW and mortality in cardiovascular diseases may be related to the underlying inflammation [39]. In a cross-sectional study in which the authors examined the laboratory parameters of 3,845 adult patients, Lippi et al. showed that high RDW values were associated with ESR and high-sensitivity CRP, which are inflammatory markers [10]. In our study group, we found a correlation between the CRP and RDW values (Figure 1).

Fibrinogen is also one of the acute phase reactants that increases during the FMF attacks and is expected to return to normal values during the attack-free periods [4,40]. Similarly, we detected a correlation between CRP and fibrinogen levels in FMF patients (Figure 2).

In the literature, there are studies showing that the NLR is important in determining the risk of developing amyloidosis and ongoing inflammation in patients with FMF during attack free-periods [5,9,41]. In our study, NLR was found to be the same in the healthy and FMF patient groups. However, NLR was higher in patients with amyloidosis than those without it.

When only the FMF patients with or without amyloidosis were considered, in patients who received anti-IL-1 therapy, the fibrinogen level, RDW, and NLR, as well as inflammatory parameters such as ESR and CRP, were higher in patients who had amyloidosis than those who had not. Besides, when we evaluated whether the fibrinogen, NLR, and RDW values could be used as a diagnostic marker for amyloidosis positivity, we found a cut-off value of ≥2.97, sensitivity of 75.00%, and specificity of 65.40% for fibrinogen, a cut-off value of ≥2.16, sensitivity of 70.00%, and specificity of 43% for NLR, and a cut-off value of for ≥15.40, sensitivity of 75.00%, and specificity of 76.90% for RDW (Table 6). These findings demonstrate that a fibrinogen value ≥2.97, an NLR value ≥2.16, and a RDW value ≥15.40 may be used in determining the amyloidosis risk in FMF patients.

Although there are studies in the literature that state that anti-IL-1 therapy controls the acute phase reactants in FMF patients with amyloidosis [17,42], we could not observe a similar effect in our study.

It has been reported that the male gender is a risk factor in the development of amyloidosis [42,43]. In accordance with the literature, in the present study, we found that most FMF patients with amyloidosis were males and most FMF patients without amyloidosis were females.

Arthralgia/arthritis was more frequent in the nonamyloidosis group this may be related to the longer duration of anti-IL-1 therapy which may suppress joint findings in the amyloidosis-positive group.

Mutations in the MEFV gene, which encode a protein called pyrin which is essential in the innate immune system, may be associated with FMF [15,43]. A more severe course of the disease in patients with a homozygous gene mutation is well known [44]. In our study group, while the M694V homozygous gene mutation ratio was about 60% for the patients with amyloidosis, this ratio was decreased to about 40% in those without amyloidosis; a finding consistent with the literature.

The fact that our study was not a prospective, controlled study in which the drug effect was demonstrated is our main limitation. Another limitation is that we compared FMF patients receiving anti-IL-1 therapy with only healthy individuals; FMF patients who did not receive anti IL-1 therapy were not included in the study.

## 5. Conclusion

Among FMF patients who received anti-IL-1 therapy, fibrinogen, NLR, and RDW, as well as inflammatory parameters such as ESR and CRP levels, were higher in patients with amyloidosis than those without amyloidosis. Anti-interleukin-1 therapy may not sufficiently control the subclinical inflammation parameters in the amyloidosis group. Multicenter prospective controlled studies are needed to demonstrate the effects of these drugs on preventing the development of amyloidosis in FMF patients.

## Figures and Tables

**Table 1:** Comparison of the FMF patient and control groups in terms of age, gender, and laboratory values.

	FMF group (n = 46)	Control group (n = 36)	p

**Age (years), mean ± SD**	40.60 ± 11.13	41.50 ± 9.39	0.701*

**Sex, n (%)**			0.657‡^a^
Male	21 (45.7)	19 (52.8)	
Female	25 (54.3)	17 (47.2)	
Total	46 (100.0)	36 (100.0)	

**ESR (mm/h), median (min–max)**	**11 (3–106)**	8 (3**–**38)	**0.024** †

**CRP (mg/L), median (min–max)**	**3.12 (0.5–57**)	1.28 (0.5**–**8.6)	**<0.001** †

**WBC (× 1000/mL), mean ± SD**	7.59 ± 2.84	6.73 ± 1.36	0.077*

**Neutrophils (× 1000/mL), mean ± SD**	4.52 ± 2.09	4.06 ± 0.98	0.194*

**Lymphocytes (× 1000/mL), mean ± SD**	2.22 ± 0.90	2.13 ± 0.53	0.587*

**NLR, median (min–max)**	1.91 (0.86**–**7.5)	1.84 (1**–**4.45)	0.688†

**Platelets (× 1000/mL), mean ± SD**	270.00 ± 75.60	279.08 ± 64.47	0.567*

**Hemoglobin (g/dL), median (min–max)**	**13.1 (8–17.3)**	14.5 (9**–**17.3)	**0.003** †

**RDW, median (min–max)**	**15 (12–21.5)**	13.3 (12.4–24)	**<0.001** †

**MPV, mean ± SD**	8.30 ± 1.24	8.25 ± 0.86	0.816*

CRP: C-reactive protein, ESR: Erythrocyte sedimentation rate, FMF: Familial Mediterranean fever, MPV: Mean platelet volume, NLR: Neutrophil-to-lymphocyte ratio, RDW: Red cell distribution width, SD: Standard deviation, WBC: White blood cell.

* T-test,

† Mann-Whitney U test,

‡ Chi-square test (Fisher’s exact test^a^)

Significant p values are written in bold.

**Table 2:** Correlation between the CRP, NLR, RDW, and MPV levels in FMF patients.

		CRP	NLR	RDW	MPV
**NLR**	Correlation coefficient (r)	0.371*			
	p	0.011			
	N	46			
**RDW**	Correlation coefficient (r)	0.541†	0.307*		
	p	**<0.001**	.038		
	N	46	46		
**MPV**	Correlation coefficient (r)	0.124	0.064	0.169	
	p	0.412	0.671	0.261	
	N	46	46	46	
**Fibrinogen**	Correlation coefficient (r)	0.439†	0.422†	0.362*	0.102
	p	**0.002**	**0.004**	**0.013**	0.498
	N	46	46	46	46

CRP: C-reactive protein, MPV: Mean platelet volume, NLR: Neutrophil-to-lymphocyte ratio, RDW: Red cell distribution width.

* Correlation is significant at the 0.05 level (two-tailed),

† Correlation is significant at the 0.01 level (two-tailed)

Significant p values are written in bold.

**Table 3:** Comparison of the FMF patients according to amyloidosis presence in terms of demographic and clinical data.

	Amyloidosis (+) (mean ± SD) (n = 20)	Amyloidosis (−) (mean ± SD) (n = 26)	p

**Age** (**years**), **median (min–max)**	40 (24**–**61)	41 (21**–**62)	0.807*

**BMI (kg/m** **^2^** **), median (min–max)**	23.9 (16.3**–**34.7)	27.1 (19.03**–**34.8)	0.099*

**Age at diagnosis (years), median (min–max)**	25 (5**–**50)	30 (6**–**52)	0.557*

**Duration of disease (months), median (min–max)**	10.5 (5**–**29)	10 (3**–**25)	0.721*

**Colchicine dose administered, median (min–max)**	1 (0.5**–**2)	1 (0.5**–**2)	0.185*

**Duration of IL-1 antagonist therapy (months), median (min–max)**	**69.5 (23–85)**	31 (5**–**84)	**0.005** *

**Gender, n (%)**			**<0.001** † ^a^
Male	**16 (80.0)**	5 (19.2)	

Female	4 (20.0)	**21 (80.8)**	

**Family history, n (%)**			0.365†^a^
Absent	5 (25.0)	10 (38.5)	

Present	15 (75.0)	16 (61.5)	

**Consanguineous marriage, n (%)**			0.187†^a^
Absent	12 (60.0)	21 (80.8)	
Present	8 (40.0)	5 (19.2)	

**Genetic mutation, n (%)**			0.307†
Normal	1 (5.0)	3 (11.5)	

M694V homozygous	12 (60.0)	10 (38.5)	

M694V heterozygous	2 (10.0)	2 (7.7)	

M694V/E148Q	0 (0.0)	4 (15.4)	

Other	5 (25.0)	7 (26.9)	

**Fever, n (%)**			0.726†^a^
Absent	5 (25.0)	5 (19.2)	

Present	15 (75.0)	21 (80.8)	

**Erysipelas-like erythema, n (%)**			0.749†^a^
Absent	15 (75.0)	18 (69.2)	

Present	5 (25.0)	8 (30.8)	

**Peritonitis, n (%)**			0.622†^a^
Absent	1 (5.0)	3 (11.5)	

Present	19 (95.0)	23 (88.5)	

**Pleurisy, n (%)**			0.302†^a^
Absent	17 (85.0)	18 (69.2)	

Present	3 (15.0)	8 (30.8)	

**Arthralgia/arthritis, n (%)**			**0.042** † ^a^
Absent	14 (70.0)	10 (38.5)	

Present	**6 (30.0)**	**16 (61.5)**	

**Myalgia, n (%)**			0.129†^a^
Absent	15 (75.0)	13 (50.0)	

Present	5 (25.0)	13 (50.0)	

BMI: Body mass index, SD: Standard deviation.

* Mann-Whitney U Test,

† Chi-square test (Fisher’s exact test^a^)

Significant p values are written in bold.

**Table 4:** Comparison of various laboratory values of FMF patients according to the presence of amyloidosis.

	Amyloidosis (+), median (min–max) (n = 20)	Amyloidosis (−), median (min–max) (n = 26)	p
**Fibrinogen (g/L)**	**3.34 (1.14–18.6)**	2.73 (1.67**–**5.09)	**0.018** *
**ESR (mm/h)**	**19.5 (3–106)**	8 (3**–**47)	**0.039** *
**CRP (mg/L)**	**7.1 (1.03–57)**	2.67 (0.5**–**26)	**0.022** †
**WBC (× 1000/mL)**	**8.21 (4.13–17.2)**	6.67 (2.67**–**10.7)	**0.019** *
**Neutrophils (× 1000/mL)**	**4.76 (2.56–11.24)**	3.89 (1.34**–**8.63)	**0.008** *
**Lymphocytes (× 1000/mL)**	2.1 (0.97**–**5.05)	2.23 (0.92**–**4.31)	0.938*
**NLR**	**2.31 (1.45–5.97)**	1.7 (0.86**–**7.5)	**0.007** *
**Platelets (× 1000/mL)**	255 (162**–**435)	292.5 (150**–**354)	0.618*
**Hemoglobin (g/dL)**	12.2 (8**–**17.2)	13.7 (8**–**17.3)	0.227*
**RDW**	**16.45 (13.6–21.5)**	13.9 (12**–**18.6)	**<0.001** *
**MPV**	8 (7.1**–**11.3)	7.95 (6.1**–**11.4)	0.610*

CRP: C-reactive protein, ESR: Erythrocyte sedimentation rate, MPV: Mean platelet volume, NLR: Neutrophil-to-lymphocyte ratio, RDW: Red cell distribution width, WBC: White blood cell.

* Mann-Whitney U test,

† Chi-square test

Significant p values are written in bold.

**Table 5:** Multivariate logistic regression analysis to identify the risk factors that increase the risk of amyloidosis in FMF patients.

	SE^†^	OR^†^	95% CI^†^	*p*
**ESR**	0.038	1.087	1.008**–**1.172	**0.029** *
**Sex (male)**	1.673	101.571	3.822**–**2699.087	**0.006** *
**Arthralgia/arthritis (present)**	1.958	51.131	1.101**–**2374.949	**0.045** *
**RDW**	0.392	2.374	1.102**–**5.113	**0.027** *
**Constant**	8.369	0.000		0.014

SE=Standard error, OR=Odds Ratio, CI: Confidence interval, ESR: Erythrocyte sedimentation rate, RDW: Red cell distribution width

* Multivariate binary logistic regression (Backward: LR), (Omnibus tests of model coefficients <0.001, Nagelkerke’s R-square = 0.732, Hosmer-Lemeshow test = 0.832)

Significant p values are written in bold.

**Table 6:** ROC curve analysis and cut-off values for fibrinogen, RDW, and NLR as a diagnostic marker for amyloidosis positivity in FMF patients.

	Diagnostic test					ROC curve			p
	Cut-off Value	Sensitivity	Specificity	PPD	NPD	AUC	Standard error	95% CI	
**Fibrinogen**	≥**2.97**	75.00	65.40	52.20	47.80	**0.705**	0.080	0.548–0.862	**0.018** *
**NLR**	≥**2.16**	70.00	43.50	77.00	56.50	**0.734**	0.075	0.586–0.881	**0.007** *
**RDW**	≥**15.40**	75.00	76.90	45.70	54.30	**0.827**	0.060	0.710–0.944	**<0.001** *

AUC: Area under the curve, CI: Confidence interval, NLR: Neutrophil-to-lymphocyte ratio, NPD: Negative predictive value, PPD: Positive predictive value, ROC: Receiver operating characteristic. RDW: Red cell distribution width.

* ROC curve analysis

Significant p values are written in bold.
